# Erythrocytes enhance oxygen-carrying capacity through self-regulation

**DOI:** 10.3389/fphys.2025.1592176

**Published:** 2025-05-16

**Authors:** Ying Xu, Zhangjie Yu, Hanxuan Liu, Xiaohan Bian, Weiliang Tang

**Affiliations:** ^1^ School of Medicine, ShaoXing University, Shaoxing, Zhejiang Province, China; ^2^ Department of Cardiology, Shaoxing People's Hospital, Shaoxing, Zhejiang, China

**Keywords:** erythrocytes, hypoxia, nitric oxide, adenosine, βCys93, drug delivery systems, RBC eNOS, CYB5R3

## Abstract

Once considered passive carriers of oxygen, erythrocytes are now understood to play active roles in regulating oxygen homeostasis and redox balance. This review examines the molecular mechanisms through which red blood cells adapt to hypoxic conditions, including nitric oxide (NO)-driven changes in membrane properties, βCys93-dependent S-nitrosylation, adenosine-induced activation of glycolysis, and the development of hypoxic memory via eENT1 degradation. Enzymes such as RBC eNOS, CYB5R3, and G6PD are essential for maintaining NO availability and redox balance by controlling redox state and NADPH synthesis. In addition to their role in gas transport, erythrocytes contribute to intercellular communication, retain organelle remnants under pathological conditions, and are being explored as platforms for drug delivery. Progress in nanotechnology and gene editing has expanded their clinical applications. These findings present erythrocytes as adaptable, multifunctional cells that connect cellular metabolism, vascular biology, and translational research.

## 1 Introduction

Erythrocytes, or red blood cells (RBCs), are the most abundant cell type in the human body and serve as the primary transporters of oxygen. This role is enabled by hemoglobin (Hb), a tetrameric protein consisting of two alpha and two beta subunits. Each subunit contains a globin chain and a heme group centered on an iron ion (Fe^2+^) that reversibly binds oxygen. Hemoglobin exhibits cooperative binding, once one oxygen molecule binds, its affinity for additional oxygen molecules increases (Szczesny-Malysiak et al., 2020). This property allows erythrocytes to efficiently load oxygen in the lungs and release it in peripheral tissues as required.

Oxygen is fundamental to nearly all cellular processes, and a deficiency, hypoxia, can impair development, damage tissues, and ultimately lead to organ failure. Hypoxia may arise under physiological conditions, such as high-altitude exposure, or from pathological states including anemia, cardiovascular disease, chronic obstructive pulmonary disease (COPD), neurodegenerative diseases, and chronic kidney disease (Storz and Bautista, 2022). In physiological hypoxia, adaptive mechanisms such as vasodilation and increased blood flow can help restore balance. However, in pathological hypoxia, these responses are often disrupted, resulting in sustained oxygen deficiency at the tissue level.

Conventional treatments for hypoxia, including oxygen therapy and intravenous oxygen carriers, can increase blood oxygen content but often fail to address underlying issues in microvascular perfusion. Raising oxygen levels in the bloodstream does not ensure that tissues receive sufficient oxygen for their metabolic needs, since effective delivery relies on finely regulated microcirculatory flow rather than oxygen content alone (Mallat et al., 2022). Given this complexity, a deeper understanding of how erythrocytes actively participate in local oxygen regulation is essential. This review examines how red blood cells adjust their oxygen transport capacity under hypoxic stress and investigates the molecular processes by which they detect and respond to changes in oxygen availability.

## 2 Mechanisms for improving hypoxia

To meet the oxygen demands of tissues, particularly during physiological stress or disease, erythrocytes must do more than passively transport oxygen; they must adapt both structurally and functionally. A range of regulatory mechanisms enables red blood cells to adjust their oxygen delivery during hypoxia. These include the modulation of nitric oxide (NO) signaling, alterations in membrane deformability, and biochemical changes initiated by specific amino acid residues in hemoglobin. This section examines how erythrocytes detect and respond to hypoxic conditions through molecular pathways that support oxygen delivery and vascular regulation.

### 2.1 Nitric oxide: a key regulator of vascular tone and oxygen transport

NO is a small yet highly reactive molecule that plays a central role in maintaining vascular equilibrium and managing oxygen distribution. Produced from L-arginine by nitric oxide synthase (NOS), NO contributes to several physiological functions, such as vasodilation, regulation of blood pressure, and control of blood flow. By activating soluble guanylate cyclase (sGC), NO elevates cyclic guanosine monophosphate (cGMP) levels, which relaxes smooth muscle and facilitates vasodilation (Wu et al., 2021).

There are three main NOS isoforms: endothelial (eNOS), inducible (iNOS), and neuronal (nNOS). Of these, eNOS generates nearly half of the circulating NO and is primarily responsible for sustaining vascular tone under baseline conditions. iNOS is activated during inflammatory responses or hypoxia and produces large amounts of NO, which can lead to oxidative stress if not properly controlled (Guo et al., 2023). nNOS is mainly expressed in the nervous system and plays a role in neurotransmission and synaptic plasticity.

In the bloodstream, NO is rapidly captured by hemoglobin, forming nitrosyl-hemoglobin (HbNO) (Dent et al., 2021). While this reaction reduces the systemic availability of free NO, erythrocytes can still participate in NO-related signaling. Rather than serving solely as NO sinks, red blood cells may locally regulate its actions, influencing both vascular tone and oxygen unloading. This becomes especially relevant in the microcirculation, where capillaries lack smooth muscle and conventional NO–cGMP signaling pathways do not operate. This alternative regulatory route highlights the distinctive role erythrocytes play in managing perfusion during hypoxic stress.

#### 2.1.1 NO enhances erythrocyte deformability

Erythrocyte deformability, the capacity of red blood cells to alter shape as they traverse narrow capillaries, is vital for effective oxygen delivery. This flexibility arises from the coordinated behavior of membrane lipids, integral and peripheral proteins, and the underlying cytoskeletal network. In contrast to artificial lipid bilayers, erythrocyte membranes exhibit low bending rigidity, largely due to their heterogeneous lipid content and a dynamic cytoskeletal framework. These structural features allow red blood cells to maintain both flexibility and oxygen-carrying capacity (Himbert et al., 2022).

Prolonged hypoxia, however, can compromise this property. Elevated reactive oxygen species (ROS) drive lipid peroxidation, disrupting membrane structure and increasing stiffness (Hay et al., 2022). Reduced deformability impairs oxygen transport, promotes chronic inflammation, and contributes to microvascular blockage and tissue injury. Sickle cell disease (SCD) exemplifies this process; a point mutation in the β-globin gene leads to hemoglobin polymerization under low oxygen conditions, generating stiff, sickle-shaped cells that obstruct blood flow and provoke ischemic damage (Wang and Zennadi, 2021).

NO helps counteract oxidative stress and is involved in the management of hypoxia-related disorders by improving erythrocyte deformability, thereby supporting more efficient oxygen delivery and better clinical outcomes (Anastasiadi et al., 2024a). It promotes membrane fluidity and shields cells from oxidative injury. A key mechanism involves the formation of S-nitrosothiols (SNOs) through NO interaction with protein thiol groups, such as βCys93 on hemoglobin or free cysteine residues. These SNOs stabilize protein conformation or influence enzyme function through post-translational modifications, improving the red blood cell’s ability to withstand hypoxic stress (Dent and DeMartino, 2023).

Various protein kinases and phosphatases tightly regulate erythrocyte signal transduction, allowing rapid responses to hypoxia, metabolic shifts, oxidative stress, and shear forces. Among these regulatory elements, the Band 3 anion transport protein is one of the most abundant transmembrane proteins in erythrocytes and plays a central role in maintaining cell function. Band 3 is crucial for preserving erythrocyte deformability during passage through narrow capillaries. Its activity is dynamically modulated by phosphorylation and dephosphorylation, primarily mediated by protein tyrosine kinases (PTKs) and protein tyrosine phosphatases (PTPs), respectively. The phosphorylation state of Band 3 influences its interaction with ankyrin, a cytoskeletal anchor. Phosphorylation can weaken the ankyrin–Band 3 interaction, destabilizing the cytoskeleton and impairing erythrocyte mechanical properties (Cilek et al., 2024).

**FIGURE 1:**
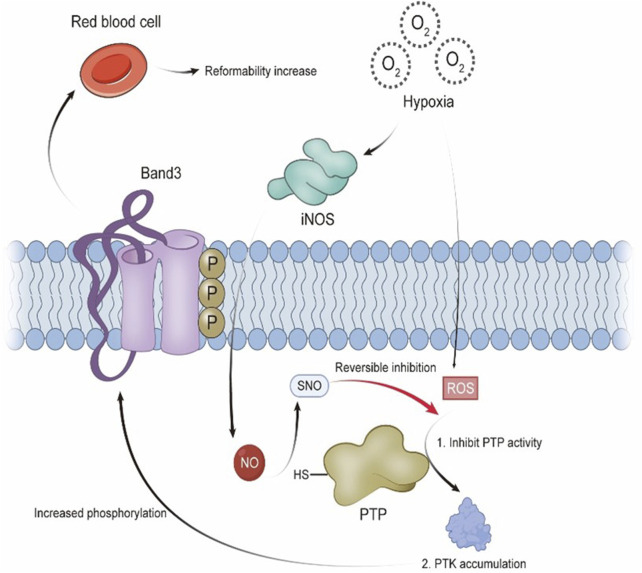
Under hypoxic conditions, reactive oxygen species (ROS) inhibit the activity of protein tyrosine phosphatases (PTPs), leading to increased phosphorylation of Band 3 and reduced erythrocyte deformability. Nitric oxide (NO) restores PTP activity via S-nitrosothiol (SNO) modification, counteracting the oxidative effects on Band 3 phosphorylation.

In human erythrocyte membranes, PTPs are positioned near Band 3 and generally show high activity to prevent excessive tyrosine phosphorylation. However, under hypoxic conditions, increased ROS lead to the oxidation of cysteine residues, inhibiting PTP function (Salmeen and Barford, 2005). As a result, PTK activity becomes dominant, leading to increased Band 3 phosphorylation, reduced deformability, and altered blood rheology. Notably, iNOS expression is elevated in hypoxia, raising NO production (Sun et al., 2006). As NO levels increase, PTP activity is partly restored, counteracting the inhibitory influence of ROS and reducing the hypoxia-driven rise in tyrosine phosphorylation on Band 3. Through this mechanism, NO contributes to maintaining the structural integrity of erythrocyte membrane proteins and supports membrane flexibility. These effects are essential for sustaining normal blood flow in the microcirculation and preventing hemorheological alterations that may arise under hypoxic conditions. (See Figure 1 for a schematic overview.)

RBCs have traditionally been viewed solely as carriers of respiratory gases, yet accumulating evidence now shows that they contain functional eNOS capable of synthesizing NO (LoBue et al., 2023). RBC eNOS is situated near the membrane cytoskeleton and shares key features with endothelial eNOS, including its dependence on L-arginine, oxygen, and the cofactor tetrahydrobiopterin (BH4) for NO production. The reducing equivalents required for this reaction are supplied by NADPH, generated through the pentose phosphate pathway within the RBC (Dore et al., 2021). Despite lacking nuclei and organelles, RBCs retain both eNOS protein and components of its regulatory system. Under physiological conditions, mechanical stimuli such as shear stress or deformation can activate RBC eNOS. *In vitro* experiments have shown that shear stress increases phosphorylation at Ser1177 on eNOS, a known activation site, and leads to elevated NO production (Nagarajan et al., 2016). A portion of the NO synthesized by RBC eNOS can diffuse into surrounding tissues or be converted into nitrite under hypoxic conditions, supporting vasodilation and contributing to the regulation of blood flow in oxygen-deprived areas (LoBue et al., 2023). As such, RBC-derived NO has emerged as a noncanonical yet vital contributor to circulating nitric oxide. Recent *in vivo* studies have further defined the physiological role of RBC eNOS. Mice with RBC-specific deletion of eNOS show no change in resting coronary blood flow but exhibit elevated systemic blood pressure, indicating that RBC-derived NO contributes to maintaining vascular tone (Leo et al., 2021). In ischemic heart injury models, mice lacking eNOS in RBCs develop larger infarcts and more severe cardiac dysfunction, whereas restoring eNOS expression in RBCs reduces myocardial damage (Cortese-Krott et al., 2022). These findings demonstrate that RBCs can generate bioactive NO internally and that this function is significant for cardiovascular regulation and injury response (Cortese-Krott et al., 2022).

Hemoglobin, the most abundant protein in RBCs, engages in dynamic interactions with NO, forming a range of redox-related derivatives, including methemoglobin (MetHb) and HbNO. Cytochrome b5 reductase 3 (CYB5R3), a NADH-dependent enzyme, is responsible for maintaining hemoglobin in its functional ferrous (Fe^2+^) state. It catalyzes the reduction of ferric iron (Fe^3+^) in MetHb back to Fe^2+^, preserving hemoglobin’s oxygen-carrying ability and preventing NO-induced inactivation (Rahaman et al., 2017; Hall et al., 2022). Through continuous redox cycling, CYB5R3 keeps hemoglobin in an active state and shields erythrocytes from oxidative damage caused by NO and ROS (Ma et al., 2023). Beyond supporting oxygen transport, CYB5R3 also shapes NO signaling by regulating hemoglobin’s redox state. During NO production by eNOS, hemoglobin reacts with NO to form transient species like MetHb and HbNO. CYB5R3 quickly converts these forms back to functional hemoglobin, helping stabilize the local NO environment within RBCs (Kuhn et al., 2017). Similar regulatory processes have been identified in arterial endothelial cells (Farah et al., 2018). Straub et al. showed that eNOS, hemoglobin α, and CYB5R3 form a functional complex in endothelial cells, where CYB5R3-driven reduction of heme iron influences NO availability (Katona et al., 2023). In the Fe^2+^ state, hemoglobin binds NO tightly, limiting its release, while the Fe^3+^ form permits NO to diffuse and contribute to vasodilation. Given the high hemoglobin content in RBCs, this mechanism likely operates in parallel, positioning CYB5R3 as a central regulator of both hemoglobin activity and NO signaling dynamics. Under localized hypoxic or oxidative stress, if the rate of hemoglobin oxidation exceeds CYB5R3’s reductive capacity, NO or its derivatives, such as S-nitrosothiols, may be released. In this setting, CYB5R3 functions not only as a preserver of hemoglobin activity but also as a responsive “regulatory valve” controlling NO signaling within RBCs.

CYB5R3 also plays a wider role in sustaining redox balance. In CYB5R3-deficient conditions, MetHb builds up, glutathione (GSH) levels rise in response to oxidative stress, and RBCs display morphological changes such as microcytosis, acanthocytosis, and increased membrane fragility (Jenni et al., 2023). Interestingly, some animal models with CYB5R3 deficiency exhibit increased RBC deformability, possibly due to compensatory alterations in cytoskeletal structure (Hertz et al., 2017). These findings further highlight the diverse roles of CYB5R3 in maintaining both the biochemical stability and physical properties of erythrocytes.

Due to the lack of mitochondria, RBCs depend on the pentose phosphate pathway (PPP) to produce reducing equivalents in the form of NADPH. This pathway is initiated by glucose-6-phosphate dehydrogenase (G6PD), the rate-limiting enzyme essential for maintaining redox balance in the cell (Dore et al., 2021). NADPH supports several key functions in RBCs: it supplies reducing power for RBC eNOS-mediated NO production, keeps tetrahydrobiopterin (BH_4_) in its active form, and fuels the glutathione-based antioxidant defense system. When G6PD activity is deficient, NADPH levels fall, increasing the likelihood of RBC eNOS uncoupling, where the enzyme generates ROS instead of NO (Snezhkina et al., 2019). Simultaneously, the weakened glutathione system loses its ability to neutralize peroxides, which intensifies oxidative stress. This not only promotes No scavenging by superoxide but also contributes to the formation of peroxynitrite (ONOO^−^) (Spinelli et al., 2024). G6PD controls the NO pathway in RBCs at two main levels: (1) directly, by regulating NADPH supply, which affects both eNOS activity and its coupling status; and (2) indirectly, by powering antioxidant defenses that protect NO and hemoglobin from oxidative damage. When G6PD function is impaired, RBCs accumulate MetHb and ROS. If NADH is also in short supply, CYB5R3’s reductive activity may decline, causing a combined loss of hemoglobin’s oxygen-carrying function and its capacity to buffer NO (Fujii et al., 2021). Under extreme oxidative stress, exogenous reducing agents such as methylene blue may partly restore redox balance by enabling NADPH-driven alternative reduction of MetHb, compensating for deficiencies in both G6PD and CYB5R3. Clinically, individuals with G6PD deficiency are particularly vulnerable to methemoglobinemia and hemolytic episodes, especially during infections or other oxidative stressors. This susceptibility reflects a broader disruption in redox control and NO signaling (Hall et al., 2022). In parallel, G6PD-deficient mice exhibit lower baseline NO levels, increased oxidative burden, and impaired vascular responses, especially under stress conditions like a high-fat diet. These mice also develop elevated blood pressure and show signs of endothelial dysfunction (Dore et al., 2021).

In summary, G6PD plays a central role in shaping the redox landscape of RBCs. Its control over NADPH production determines eNOS activity, supports CYB5R3 function, and maintains antioxidant defenses. Together, these elements form a coordinated network that regulates NO signaling and the redox state of hemoglobin within erythrocytes.

Under hypoxic conditions, calcium (Ca^2+^) and magnesium (Mg^2+^) are key to preserving erythrocyte membrane deformability. During acute hypoxia, adenosine triphosphate (ATP) binds to hemoglobin, lowering its oxygen affinity to promote oxygen release to peripheral tissues. However, as energy production becomes compromised, intracellular ATP levels drop, impairing the activity of Ca^2+^-ATPase on the erythrocyte membrane. This disruption affects ion transport, preventing the proper extrusion of Ca^2+^. Accumulation of intracellular calcium raises osmotic pressure, leading to water retention and making it more difficult for erythrocytes to maintain their usual biconcave shape. In addition, calcium fluctuations weaken the interaction between hemoglobin and the membrane, encouraging hemoglobin to shift into the cytoplasm (Livshits et al., 2023).

Mg^2+^ acts as a cofactor for Ca^2+^-ATPase, boosting pump activity to help maintain low intracellular Ca^2+^ levels and preventing membrane calcification (Lima and Fock, 2020). Beyond its role in ion regulation, Mg^2+^ influences the assembly and organization of the erythrocyte cytoskeleton, which is primarily composed of actin and spectrin. This cytoskeletal network is essential for maintaining mechanical flexibility. As erythrocytes move through narrow microvessels, they continuously adjust their shape through cytoskeletal remodeling. Mg^2+^ supports this adaptability by regulating the activity of cytoskeletal proteins, helping to preserve structural integrity and functional plasticity under hypoxic stress (Vakhrusheva et al., 2022).

#### 2.1.2 The role of βCys93 in counteracting tissue hypoxia

Among the conserved amino acids in hemoglobin, the cysteine at position 93 on the β-globin chain (βCys93) plays a key role in the physiological response to low oxygen availability (Jensen, 2009). While histidine helps regulate the redox state of the heme iron and phenylalanine supports the protein’s structural integrity, βCys93 is especially important for NO-mediated vascular control. This function is primarily driven by S-nitrosylation, a reversible modification where NO binds to protein thiol groups, forming S-nitrosothiols (SNOs) (Aboalroub and Al, 2024). These SNOs influence protein activity, enzyme regulation, and cellular signaling pathways. In red blood cells, βCys93 serves as a main site for this modification, directly linking hemoglobin to NO storage and release.

During hypoxic stress, hemoglobin undergoes a conformational shift that exposes βCys93, allowing SNO-hemoglobin (SNO-Hb) to form. This NO-loaded form of hemoglobin can release NO, which is then transferred to targets such as Band 3 on the erythrocyte membrane or to thiol-containing molecules in the endothelium (Su et al., 2020). The effect is localized vasodilation, improving oxygen delivery to tissues with the greatest need.

SNO derived from erythrocytes also plays a key role in communicating with endothelial cells during hypoxia, promoting vasodilation through NO release. This mechanism is vital for ensuring oxygen reaches hypoxic regions efficiently, emphasizing βCys93’s contribution to oxygen homeostasis under stress (Premont et al., 2020).

The importance of βCys93 has been demonstrated in animal models. Mice engineered to carry the βCys93Ala mutation, which eliminates the thiol group, are unable to generate SNO-Hb and show impaired regulation of blood flow during hypoxia (Hausladen et al., 2022). Even under normoxic conditions, these mice display cardiac stress, including reduced T-wave amplitude on electrocardiograms, suggesting a protective role for βCys93 in maintaining heart function (Sun et al., 2019).

However, excessive SNO-Hb formation can be harmful. High levels of NO signaling under normoxia or hyperoxia may interfere with oxygen-sensing mechanisms and disrupt physiological responses to hypoxia (Zhang et al., 2015). These observations highlight the importance of precise NO regulation through βCys93, allowing red blood cells to detect and respond to oxygen deficiency in real time without overcompensating.

### 2.2 Adenosine-mediated signaling in hypoxia adaptation

Adenosine is a small molecule with strong biological activity, especially under hypoxic conditions. It has recently gained attention for its role in helping the body respond to low oxygen not only by promoting vasodilation but also by regulating the interactions between red blood cells and the vascular system (Zhang et al., 2021).

During sustained oxygen deficiency, extracellular adenosine levels rise sharply. This increase is driven by the sequential actions of two enzymes: CD39, which converts ATP to AMP, and CD73, which converts AMP into adenosine. The resulting spike in adenosine activates the A2B adenosine receptor (ADORA2B), which is present on both red blood cells and endothelial cells. Once engaged, ADORA2B triggers a series of adaptive responses. These include vasodilation to boost blood flow and improved coordination between oxygen delivery and metabolic demand. Adenosine also helps shield tissues by suppressing inflammation and limiting oxidative stress, both of which are common during hypoxic injury. This pathway is especially relevant in high-altitude environments. Individuals living or training at elevation often show increased CD73 activity, which maintains higher adenosine levels (Boussuges et al., 2022). In contrast, people lacking functional CD73 are more prone to organ damage during hypoxic stress, particularly in the heart, brain, and kidneys (Ndzie Noah et al., 2024). These findings identify adenosine not only as a signaling molecule but also as a critical component of the body’s adaptive defense against oxygen shortage.

#### 2.2.1 S1P enhances oxygen release via glycolysis

A key downstream effect of adenosine signaling in red blood cells involves the lipid mediator sphingosine-1-phosphate (S1P). While S1P is best known for its roles in immune regulation and vascular stability, it has emerged as a significant factor in controlling hemoglobin’s oxygen release, particularly under hypoxic stress. S1P is synthesized within cells through a multistep pathway: ceramide is broken down into sphingosine, which is then phosphorylated by sphingosine kinase 1 (SphK1) to generate S1P. Under conditions such as inflammation or oxygen deprivation, SphK1 activity rises, leading to increased S1P concentrations in red blood cells (Sassoli et al., 2019).

This rise in S1P is not an isolated response. In animal models of chronic hypoxia, such as high-altitude polycythemia, Yu et al. reported concurrent increases in S1P, CD73, adenosine, and 2,3-bisphosphoglycerate (2,3-BPG) (Yu et al., 2024). These molecules form a coordinated network that enhances oxygen unloading from hemoglobin. Adenosine activates ADORA2B receptors, which stimulate SphK1 activity, promote glycolysis, and boost 2,3-BPG production, a molecule that lowers hemoglobin’s affinity for oxygen. S1P amplifies this effect by binding to hemoglobin complexes associated with 2,3-BPG, pushing them toward the T-state, a structural configuration favoring oxygen release (Sun et al., 2017). In some cases, S1P may even physically block oxygen from accessing the heme group, reinforcing the shift toward unloading. However, this mechanism has its drawbacks. In diseases such as SCD, elevated S1P and 2,3-BPG levels can shift hemoglobin too far toward deoxygenation, promoting polymerization and red cell sickling. These rigid, misshapen cells are prone to rupture and obstruct capillaries, impairing blood flow. Additionally, the shift toward glycolysis can suppress antioxidant defenses, leaving cells more exposed to oxidative stress. These findings illustrate the dual role of S1P: it enhances oxygen release under controlled conditions but, when dysregulated, contributes to red blood cell dysfunction and disease progression.

#### 2.2.2 AMPK activation enhances 2,3-BPG mutase phosphorylation

Red blood cells must rapidly adjust their internal metabolism to maintain effective oxygen delivery during hypoxia. One of the key molecular sensors driving this adaptation is AMP-activated protein kinase (AMPK). Often described as the cell’s “fuel gauge,” AMPK is activated when energy levels fall, particularly when the AMP to ATP ratio increases, an event common under low oxygen conditions.

AMPK serves as a central link between cellular energy status and oxygen transport in erythrocytes. When adenosine levels rise during hypoxia, they activate ADORA2B receptors, which in turn stimulate AMPK activity (Peng et al., 2019). Once activated, AMPK enhances the function of 2,3-bisphosphoglycerate mutase (2,3-BPGM), the enzyme responsible for producing 2,3-bisphosphoglycerate (2,3-BPG), a molecule that decreases hemoglobin’s oxygen affinity and promotes oxygen release to tissues. AMPK also influences another step in the glycolytic pathway by promoting phosphorylation of phosphoglycerate mutase (PGAM), steering more glycolytic intermediates toward the synthesis of 2,3-BPG rather than proceeding through the standard ATP-generating route (Wu et al., 2023). As a result, red blood cells shift their metabolic focus, not toward producing energy, but toward maximizing oxygen unloading. This rerouting may appear counterintuitive, but it reflects the unique priorities of erythrocytes, which do not require mitochondria to function but must respond quickly to oxygen fluctuations. Through AMPK, red blood cells can detect hypoxic stress and rapidly reprogram glycolysis to improve oxygen release, helping to preserve tissue oxygenation under challenging conditions.

#### 2.2.3 Degradation of eENT1 and the induction of hypoxic memory in erythrocytes

Adaptation to hypoxia is not limited to immediate responses, it can also involve a form of cellular memory that allows the body to react more efficiently to future oxygen deprivation. In red blood cells, this memory is linked to the regulation of equilibrative nucleoside transporter 1 (eENT1), which plays a key role in controlling adenosine levels (Song et al., 2017).

Under normoxic conditions, eENT1 facilitates the bidirectional transport of adenosine across the erythrocyte membrane, helping to maintain a balance between intracellular and extracellular concentrations. However, when oxygen levels fall, adenosine signaling through the ADORA2B receptor initiates a sequence of phosphorylation, ubiquitination, and eventual degradation of eENT1. As a result, adenosine uptake is reduced, and its concentration in the plasma increases.

This change is functionally significant. Elevated extracellular adenosine supports vasodilation, dampens inflammation, and improves oxygen delivery. By targeting eENT1 for removal, the body lifts a regulatory brake, allowing adenosine to accumulate and strengthen these protective responses. This mechanism has been observed in both animal models and humans exposed to high-altitude conditions. Following short-term exposure to extreme elevations, individuals show increased CD73 activity, which boosts adenosine production, and decreased eENT1 expression, reflecting a rapid and reversible shift in red blood cell response to hypoxia. What makes this process particularly striking is the concept of hypoxic memory. Mature erythrocytes lack nuclei and cannot synthesize new proteins, so once eENT1 is degraded, it cannot be replenished. As a result, the red blood cells remain in a hypoxia-adapted state for the rest of their lifespan. This primes them for quicker responses during subsequent hypoxic events, even if oxygen levels return to normal between exposures. However, this memory fades as aging erythrocytes are replaced by new ones with baseline eENT1 levels, unless a new hypoxic episode restarts the cycle. This system gives the body a temporary adaptive edge under stress, allowing for fine-tuned control of red blood cell function in environments like high altitude or ischemia.

This schematic depicts the regulatory network activated in red blood cells under hypoxic conditions. Hypoxia stimulates CD73 activity, resulting in increased extracellular adenosine production. Adenosine then signals through the ADORA2B receptor, triggering downstream pathways that include AMPK and PKA. AMPK activation promotes 2,3-bisphosphoglycerate (2,3-BPG) synthesis by stimulating bisphosphoglycerate mutase (BPGM) and phosphorylating phosphoglycerate mutase (PGAM), accelerating the conversion of 3-phosphoglycerate to 2-phosphoglycerate. The rise in 2,3-BPG lowers hemoglobin’s oxygen affinity, facilitating oxygen release. Simultaneously, ADORA2B-driven activation of PKA leads to the phosphorylation of SphK1, further promoting glycolytic flux and contributing to 2,3-BPG accumulation. PKA also phosphorylates ENT1, marking it for ubiquitination and subsequent proteasomal degradation. This reduces adenosine reuptake and sustains high extracellular adenosine levels. Together, these coordinated processes strengthen the erythrocyte’s adaptive response to hypoxia by enhancing its capacity to release oxygen where it is most needed.

### 2.3 ITPP reduces excessive HIF-1α expression

Under hypoxic conditions, cells activate a range of adaptive pathways to preserve function and survival. While beneficial in the short term, sustained activation of these responses can become harmful. One of the central regulators in this process is hypoxia-inducible factor-1 alpha (HIF-1α), a transcription factor that senses low oxygen levels and triggers the expression of genes involved in angiogenesis, metabolism, and cell survival. However, prolonged or excessive HIF-1α activity has been linked to inflammation, tissue remodeling, and tumor progression.

Myoinositol trispyrophosphate (ITPP), a synthetic molecule designed to modify hemoglobin’s oxygen affinity, offers a potential way to correct this imbalance. ITPP enters red blood cells and binds to the same allosteric site as 2,3-bisphosphoglycerate (2,3-BPG), shifting hemoglobin toward the T-state that favors oxygen release (Zhang et al., 2024). By improving oxygen unloading, ITPP reduces tissue hypoxia and tempers the overactivation of hypoxia-sensitive signaling.

**FIGURE 2:**
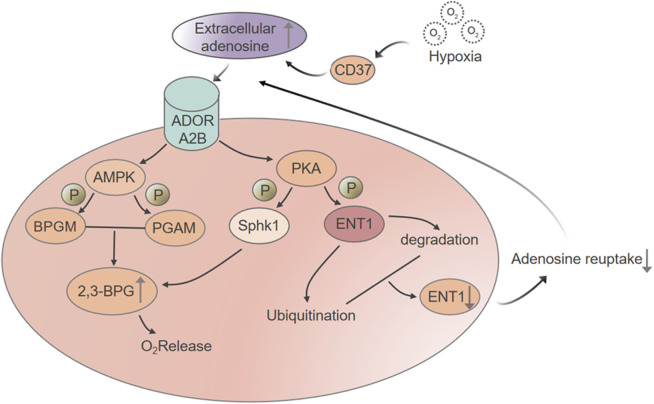
Hypoxia-driven adenosine signaling enhances 2,3-BPG synthesis and promotes oxygen release in red blood cells.

What makes ITPP distinct from conventional oxygen therapies is its precision. Instead of raising systemic oxygen levels indiscriminately, ITPP enhances oxygen delivery where it is most needed, at the tissue level. In models of heart failure, perfusion with ITPP-treated red blood cells improved cardiac performance and limited pathological remodeling. Importantly, it also led to a reduction in HIF-1α expression, indicating that restoring local oxygen availability can directly influence gene regulation (Oknińska et al., 2024). ITPP’s action appears to be pH-dependent, showing greater activity in acidic, hypoxic environments, such as ischemic myocardium, while having minimal effect in well-oxygenated, alkaline tissues (Evans et al., 2021). This adds an extra layer of selectivity, positioning ITPP as a targeted oxygen modulator. By enhancing oxygen release in a controlled and localized fashion, ITPP helps restore physiological balance and counter the harmful consequences of chronic hypoxia. This makes it a promising candidate for treating conditions such as heart failure, pulmonary hypertension, and possibly cancer, where disrupted oxygen homeostasis is a central feature (See Figure 2 for details).

## 3 Other strategies for improving hypoxia

### 3.1 Small engine, big impact: how erythrocytes maintain energy and redox balance without organelles

Erythrocyte metabolism has long been considered straightforward, relying almost exclusively on glucose breakdown via glycolysis and the PPP. This metabolic simplicity results from the absence of nuclei, mitochondria, and other organelles, which prevents erythrocytes from using oxidative phosphorylation. Consequently, glycolysis remains their sole source of ATP. Through glycolysis, glucose is enzymatically converted to pyruvate, producing ATP that supports ion transport, membrane stability, deformability, and other key functions. In parallel, the PPP provides essential antioxidant support. The oxidation of glucose-6-phosphate (G6P) in the PPP generates NADPH, a reducing equivalent that sustains glutathione (GSH) in its reduced state and protects the cell from oxidative injury (Nemkov et al., 2018). The rate-limiting enzyme in this pathway, G6PD, determines the flux through the PPP. G6PD deficiency impairs redox control and increases susceptibility to oxidative damage and hemolysis (Luzzatto et al., 2020).

During storage in blood banks, erythrocytes are exposed to ongoing oxidative stress. In this setting, the N-terminal domain of Band 3, a key membrane protein, becomes vulnerable to ROS-induced proteolysis, impairing its role in the regulation of oxygen-sensitive metabolic processes (Rogers et al., 2021). Mutations in this domain have been associated with severe hemolytic conditions requiring chronic transfusion support (Issaian et al., 2020). Hypoxic storage strategies have been proposed as a way to reduce oxidative injury during storage. Studies in mouse models have shown that erythrocytes stored under low-oxygen conditions maintain higher glycolytic activity and show increased energy metabolism, including elevated 2,3-DPG levels (Hay et al., 2022). This metabolic shift may result from increased BPGM activity at the higher pH levels typically seen in hypoxic environments. Moreover, hypoxia protects the N-terminal domain of Band 3 from ROS-mediated cleavage, preserving its ability to interact with and regulate glycolytic enzymes (Reisz et al., 2018). Under normoxic conditions, this domain binds enzymes such as GAPDH and anchors them to the membrane, keeping their activity suppressed. When oxygen levels fall, deoxygenated hemoglobin (deoxyHb) binds to Band 3, displacing these enzymes into the cytosol where they resume their function. This displacement enhances glycolytic throughput and increases 2,3-DPG synthesis, promoting oxygen release and allowing red blood cells to better meet tissue demands under hypoxic conditions (Issaian et al., 2020).

### 3.2 Nanotechnology and CRISPR-Cas9: modern innovations in hypoxia therapy

The rapid progress of nanotechnology has transformed biomedical research, particularly in drug delivery and therapeutic design. Smart drug delivery systems (DDSs) are now actively being developed for real-time biosensing and targeted treatment, features that are especially important for early cancer detection and precision-based interventions (Wang et al., 2019). A key strength of modern DDSs lies in their ability to direct diagnostic or therapeutic agents to specific tissues, organs, or cells with high accuracy. Red blood cells are well-suited as carriers in DDSs due to their biocompatibility, long circulation lifespan, and low immunogenicity. Artificial erythrocytes, built from lipid membranes and loaded with hemoglobin or functional enzymes, are designed to closely mimic the structure and function of natural red cells (Liang et al., 2021; Li et al., 2019). Among various cell-based platforms, such as bacterial ghosts, macrophages, and engineered transducer cells, RBC-based delivery systems stand out for their capacity to carry diverse therapeutic payloads. One notable advancement is the development of nano-hemoglobin-based oxygen carriers (Nano-HBOCs), which combine hemoglobin with other components through encapsulation, self-assembly, bioconjugation, or covalent attachment (Nadimifar et al., 2024). These systems improve oxygen transport and have shown promise in treating tumor hypoxia by restoring oxygen levels and sensitizing cancer cells to therapy (Zhang et al., 2023). A striking example involves the creation of hybrid nanoparticles by cross-linking hemoglobin with transferrin through disulfide bonds. These particles are capable of co-delivering photosensitizers (such as protoporphyrin IX) and chemotherapeutic agents (such as doxorubicin), using transferrin for tumor-specific targeting and hemoglobin as an internal oxygen source (Zhu et al., 2024). This multifunctional platform not only addresses tumor hypoxia but also downregulates multidrug resistance genes and HIF-1α, significantly improving the effectiveness of treatment.

Meanwhile, the CRISPR-Cas9 gene editing system, originally derived from bacterial immune defense, has emerged as a powerful tool for correcting genetic disorders. With the ability to make precise edits ranging from single-nucleotide changes to large chromosomal rearrangements, CRISPR offers new possibilities for treating inherited blood diseases (Huang et al., 2022). One of the most promising clinical targets is the *BCL11A* gene, a transcriptional repressor of γ-globin and fetal hemoglobin (HbF) production. In a study by Frangoul et al., CRISPR-Cas9 was used to disrupt an erythroid-specific enhancer of *BCL11A* in CD34^+^ hematopoietic stem and progenitor cells (HSPCs) (Frangoul et al., 2021). Following electroporation, editing efficiency exceeded 80% of alleles, with no detectable off-target mutations. The modified cells were then expanded and transplanted into patients with SCD or β-thalassemia. Post-transplantation, high levels of gene editing were observed in both bone marrow and circulating blood cells. Clinically, patients showed significant increases in HbF levels. Many with β-thalassemia achieved transfusion independence, while those with SCD experienced complete resolution of vaso-occlusive episodes. These results demonstrate the practical potential of CRISPR to reprogram gene expression and significantly improve patient outcomes. With continuing advances in delivery methods, safety, and targeting precision, CRISPR-Cas9 gene therapy is on track to become a viable, long-term treatment for hypoxia-related hematologic conditions, offering sustained relief and a better quality of life.

## 4 Future perspectives and outlook

Erythrocytes, long viewed primarily as passive oxygen carriers, are now recognized as active participants in sensing and responding to oxygen fluctuations. Recent research has identified several adaptive mechanisms through which red blood cells regulate oxygen delivery, including NO-driven changes in membrane deformability, βCys93-dependent S-nitrosylation, adenosine-mediated stimulation of glycolysis and 2,3-BPG production, and the formation of hypoxic memory via degradation of eENT1 (Peng et al., 2019; Alayash, 2021; Mikdar et al., 2021). These findings reshape our understanding of erythrocyte function and reveal new molecular targets for treating hypoxia-related diseases. In particular, allosteric hemoglobin modulators such as ITPP have shown potential in selectively enhancing oxygen release and lowering pathological HIF-1α expression, pointing to new strategies for oxygen-focused therapies (Oknińska et al., 2022).

Beyond their role in gas exchange, erythrocytes participate in a wide range of metabolic and signaling processes. These functions place them at the center of pathophysiological conditions such as hypoxia-induced injury, inflammation, neurodegeneration, aging, and cancer (Nemkov et al., 2018). Emerging studies even suggest that erythrocytes exhibit neuron-like properties: they can respond to and metabolize circulating neurotransmitters, carry out transamination reactions, and release microparticles enriched with bioactive lipids, metabolites, and glycolytic enzymes. Such features suggest that red blood cells contribute to systemic signaling and local tissue coordination in ways that have been largely overlooked (Nemkov et al., 2018). In disease contexts like systemic lupus erythematosus (SLE) and SCD, erythrocytes have been observed to retain mitochondria, an unusual feature for these cells. Additionally, the presence of DNA and various RNA species within erythrocytes hints at potential roles in gene regulation and the development of blood-based biomarkers (Anastasiadi et al., 2024b).

As drug delivery platforms, erythrocytes are attracting increasing interest. Techniques such as erythrocyte hitchhiking, where nanoparticles temporarily attach to red cell membranes, enable targeted delivery to specific organs while reducing systemic toxicity (Zhang et al., 2022). RBC-H technology is both effective and safe in delivering drugs to various organs (including the brain, liver, lung and kidney). RBC-H is also easier to target the lungs than the spleen, because the shear stress in the pulmonary capillaries removes particles from the lungs (Based on the RBC free-rider strategy design, this strategy significantly increases the absorbance of downstream organs by several orders of magnitude) (Brenner et al., 2018). One notable example is the erythrocyte-driven immunotargeting (EDIT) system, developed by Anvay Ukidve and colleagues. This approach mimics natural erythrocyte-APC interactions in the spleen to stimulate immune responses and generate prophylactic anti-tumor effects in animal models (Ukidve et al., 2020). In a related application, Li et al. developed a “supramolecular hunter” platform using Janus dendritic amphiphiles (JDA) anchored to erythrocyte membranes, which efficiently cleared toxic compounds like paraquat in both *in vitro* and *in vivo* settings (Li et al., 2020).

Despite recent progress, the full extent of erythrocytes’ systemic and multiscale role in dynamic oxygen adaptation remains only partly understood. Translating molecular discoveries into clinical practice will require stronger integration between basic research and applied medicine. Future investigations should aim to unify metabolic, structural, and signaling insights to better exploit erythrocyte plasticity for use in precision medicine. At present, most DDSs involving erythrocytes are still in preclinical stages. However, a growing number of candidates have advanced to clinical trials, where they have shown safety and early signs of therapeutic benefit. While the development of DDSs is inherently complex and time-intensive, their potential advantages, including improved targeting, lower toxicity, and extended circulation time, make them strong contenders in next-generation treatment strategies. As these technologies continue to advance, erythrocytes may no longer be viewed solely as passive oxygen transporters. Instead, they could emerge as intelligent, programmable platforms for disease detection, intervention, and systemic regulation.
